# Hyponatremia Due to Pulmonary Tuberculosis: Review of 200 Cases

**DOI:** 10.5812/numonthly.7091

**Published:** 2012-12-15

**Authors:** Nematollah Jonaidi Jafari, Morteza Izadi, Farhad Sarrafzadeh, Amir Heidari, Reza Ranjbar, Amin Saburi

**Affiliations:** 1 Health Research Center, Baqiyatallah University of Medical Sciences, Tehran, IR Iran; 2 Department of Internal Medicine, Infectious Ward, Afzalipour Educational Medical Center, Kerman University of Medical Sciences, Kerman, IR Iran; 3 Nephrology and Urology Research Center, Baqiyatallah University of Medical Sciences, Tehran, IR Iran; 4 Molecular Biology Research Center, Baqiyatallah University of Medical Sciences, Tehran, IR Iran; 5 Chemical Injuries Research Center, Baqiyatallah University of Medical Sciences, Tehran, IR Iran

**Keywords:** Tuberculosis, Hyponatremia, Causality

## Abstract

**Background:**

Pulmonary Tuberculosis (PTB) is one of the common diseases with high prevalence of mortality and morbidity in developing countries. Various complications have been reported along with PTB. The subclinical electrolyte imbalances are customary in cases with PTB.

**Objectives:**

The aim of this study was the evaluation of patients with PTB and hyponatremia.

**Patients and Methods:**

We evaluated patients with diagnosis of secondary PTB who have been admitted to Baqiyatallah hospital, Tehran, Iran from 2005 till 2010. The diagnosis of PTB was based on the appearance of acid fast bacilli in sputum smears or sputum cultures, without any evidence of miliary TB. Demographic and laboratory characteristics relative to electrolytes were recorded according inclusion and exclusion criteria.

**Results:**

The mean age was 59.22 ± 20.57 years and 91 (45.5%) patients were male. The mean serum sodium concentration was 134.54 ± 4.95 mmol/L and more than half of subjects (51%) have shown hyponatremia. The mean age difference between hyponatremic and eunatremic groups was statistically significant (61.95 versus 56.02 years) (P = 0.047). No significant relationship was found between hyponatremia and gender, anti-TB medications and co-morbidity conditions.

**Conclusions:**

In this study, an older age was suggested as an important predisposing factor for hyponatremia in patients with PTB which had been observed as less of a determinant. We recommend further evaluations for hyponatremia in patients presenting with PTB, particularly for those who are older.

## 1. Background

Hyponatremia is considered as one of the most common and important electrolyte abnormalities. Hyponatremia must be considered in all seriously ill hospitalized patients (1). Hyponatremia is defined as depletion in the serum sodium (Na) concentration to a level below 136 mmol/L and severe hyponatremia defined as serum sodium concentration lesser than 115 mmol/L which it can be considered as life-threatening condition (2, 3). The prevalence of severe hyponatremia and its non-severe form are estimated 1-4% and 15-30% of inpatients, respectively (4).

Usually, hyponatremia results from water retention secondary to an inability to match water excretion with intravenous or oral water absorption. Effective circulating volume depletion causing non-osmotic release of antidiuretic hormone (ADH) and the syndrome of inappropriate ADH secretion (SIADH) are disorders in which ADH secretion is not suppressed despite decrease in plasma osmolality. These are the two most common causes of hyponatremia (5).

The diagnosis of SIADH is established upon the exclusion of other hyponatremia etiology. This syndrome has been reported in a number of clinical conditions, such as malignancies (pulmonary, mediastinal and extrathoracic tumors), central nervous system disorders (inflammatory or demyelinating diseases, stroke and trauma), drugs (desmopressin, prostaglandin-synthesis inhibitors, phenothiazines, tricyclics and serotonin-reuptake inhibitors) and pulmonary diseases (acute respiratory failure, positive-pressure ventilation and infections) (2, 5). PTB is one of the rare pulmonary infections which can induce hyponatremia. Tuberculosis (TB) is considered as one of the common illnesses in developing countries such as Iran which can present with various clinical manifestations. TB can induce hyponatremia via several mechanisms containing local invasion to the adrenal glands (adrenal insufficiency) (6, 7), local invasion to hypothalamus or pituitary gland (8, 9), Tubercular meningitis (10-12) and inappropriate ADH secretion via pulmonary infection (13-15).

## 2. Objectives

The aim of this study was to evaluate the prevalence of hyponatremia in a large number of Iranian patients with PTB.

## 3. Patients and Methods

### 3.1. Design and Participants

We have prospectively evaluated patients with a diagnosis of secondary PTB who were admitted to the infectious wards of Baqiyatallah general hospital, Tehran, Iran from march 2005 till march 2010. Demographic and laboratory characteristics were recorded. The confirmation of secondary PTB was based on appearance of acid fast bacilli on a sputum smear or Mycobacterium tuberculosis on a sputum culture, in the absence of radiological features of miliary TB. The patients with abnormal mental status, any evidence of tubercular meningitis, edema-forming conditions, uncontrolled hyperglycemia, renal insufficiency or failure, hyperlipidemia, receiving diuretics or any medications related to SIADH or induced vasopressin release were excluded.

### 3.2. Assessment and Treatment

In addition, HIV-positive patients with diagnosis of HIV by enzyme-linked immuneabsorbent assay (ELISA) were excluded, following counselling and securing written informed consent. Any sodium or calcium (Ca) wasting condition such as renal diseases was overruled. All patients received a regular hospital diet. All patients received a typical daily regimen composed of Rifampin (R), Isoniazid (H), Pyrazinamide (Z) and Ethambutol (E) for the firsttwo months, followed by RH for the following 4-10 months (depending on the progress of the disease and treatment response based on WHO guideline). Simultaneously, blood and urine samples were gathered to determine measures of electrolytes and osmolality. Baseline blood samples and urine specimens were acquired 2 to 3 hours after breakfast. The samples were collected before any prescription intravenous fluids.

### 3.3 Statistics

Data were collected to compare the profile and laboratory characteristics of PTB patients with or without hyponatremia. The data were analyzed by using SPSS software (17th edition) and P value less than 0.05 was considered significant. Quantitative and qualitative data were reported using mean ± standard deviation (SD) and infrequency (percentage). After checking normal distribution of quantitative data, the parametric or non-parametric tests were used. For the analysis of qualitative data with normal distribution, student t-test, ANOVA, and Pearson correlation and for the abnormal distributed variables, Mann–Whitney U, Kruskal–Wallis and spearman correlation tests were used.

## 4. Results

Two hundred patients that were diagnosed and treated for active PTB have been enrolled. The mean age was 59 ± 20 years (in range of 13-102 years) and 91 (45.5%) patients were males. Females had higher mean age than males (60 ± 21 versus 57 ± 19) but this difference wasn’t statistically significant. The mean serum Na concentration was 134 ± 4 mmol/L. The female’s mean concentration of serum Na was 134 ± 4.9 vs. the male’s mean concentration of serum Na was 134 ± 5 mmol/L (P = 0.513). Of the group, 96 (48%) of the patients had normal serum Na, whereas 102 (51%) patients had hyponatremia (47.1% male vs. 52.9% female) and two (1%) patients developed hypernatremia.

There is no significant relationship between gender and hyponatremia (P = 0.670). The mean age difference between hyponatremic and eunatremic groups was statistically significant (61 versus 56 years, respectively) (P.value = 0.047). Moreover, there is no significant correlation between anti-tuberculosis medications and hyponatremia (P = 0.369). Serum Na concentration was enumerated for all different anti-TB drugs in Table 1.

**Table 1:** Serum Sodium Concentration Based on Dose of Anti-TB Drugs

Dosage	Usual Dosage, %			Max Dosage, %		
**Serum Na concentration**	**Less than 135 meq/L**	**135-145 meq/L**	**More than 145 meq/L**	**Less than 135 meq/L**	**135-145 meq/L**	**More than 145 meq/L**
**Isoniazid**	21	16.5	0	30	31.5	1
**Rifampin**	19	16	0.5	32	32	0.5
**Ethambutol**	9.5	11	0	41.5	37	1
**Pyrazinamide**	24.5	18.5	0.5	26.5	29.5	0.5

The mean corrected serum Ca concentration was 8.8 ± 0.7 mmol/L. The females mean serum Ca concentration was 8.9 ± 0.8 vs. the males mean Ca which was 8.8 ± 0.6 (P = 0.441). One hundred thirty seven (68.5%) patients had normal serum Ca concentration, whereas 59 (29.5%) patients had hypocalcaemia and 4 (2%) patients had hypercalcemia.

HTN was the most common co-morbid condition present in 46 patients (23%), while 42 patients (21%) had controlled diabetes mellitus, 8 (4%) patients had migraine headache, 1 patient had Parkinson's disease and another patient had epilepsy. There was no significant relationship between co-morbid conditions and hyponatremia (P = 0.102). Simultaneously, pneumonia was documented in 18 patients (9%), while no patients had a diagnosis of sepsis. Hospital mortality was documented in 13 patients (6.5%). No significant correlation was found between hyponatremia and mortality (P = 0.218). All of the patients with hyponatremia became eunatremic after anti-tuberculosis therapy.

## 5. Discussion

Given the findings, the prevalence of hyponatremia and hypocalcaemia obtained as 51% and 29% which is compatible with the former reports. Age was the sole variable which was different between PTB patients with and without hyponatremia. We found that hyponatremia was higher than expected in non-tubercular patients according to the previous reports. In previous studies, the prevalence of hyponatremia among inpatients, especially the ones who admitted in respiratory wards, was reported in a various range (2.48%-40%) (16, 17). This issue can be related to the type of diseases and age. As reported in several studies, patients who were admitted in children or geriatric wards, admitted in the intensive care unit or cardiac care unit, and those who admitted in the emergency department were more susceptible for hyponatremia. Furthermore hyponatremia is more to be observed in neoplastic, brain, endocrine and pulmonary diseases. The incidence of severe hyponatremia has been estimated as 1.1% in hospitalized patients whereas in that report, PTB was the most common underlying disease (24%) which is in keeping with our findings (18).

In 1969, Chung and Hubbard have noted that nearly 11% of patients with active TB (pulmonary or non-pulmonary) are affected with hyponatremia, and it is apparent that the main cause of serum sodium depletion in these patients is SIADH (19). Vorherr et al. has reported a case with PTB and hyponatremia and found antidiuretic agents in tuberculous lung tissues (14). Bryant et al. has suggested the syndrome of inappropriate secretion of antidiuretic hormone for patients with an infectious pulmonary disease such as PTB (20). Schorn et al. reported two cases of PTB and an abnormal inappropriate antidiuretic hormone level as a justifier mechanism (15). Cockcroft et al., reported a 74-year-old woman with miliary tuberculosis which had complicated by severe hyponatremia due to SIADH (21). Usalan et al. reported a case of TB who initially presented with lethargy due to hyponatremia evidently resulting from SIADH (22). Finally, Lee reported a case of PTB with refractory hyponatremia due to SIADH (13).

Although in this study we did not evaluate patients for etiology of hyponatremia, it can be beneficial if we concisely review causes of hyponatremia in patients with PTB; SIADH is a considerable complication of pulmonary infection, inflammatory and neoplastic disorders, although its prevalence and mechanism are poorly regarded. SIADH has been displayed in infectious situations such as TB. In one of the first reports, Weiss et al., reported hyponatremia in resulting from SIADH in patients with PTB (23). Then it was declared that an increased ADH level in the presence of hyponatremia in PTB cases is an indicator for ectopic ADH production. Few studies demonstrated that the ADH level was not detectable following full anti-TB therapy (13, 24).

SIADH was usually demonstrated in patients with TB and there are various causative factors for SIADH in tuberculosis. SIADH may occur following PTB, as well as tubercular meningitis. There are many reports of SIADH associated with pulmonary, miliary and central nervous system-related TB. More than 60% of the patients with tubercular meningitis may present with hyponatremia or SIADH at first presentation (25). SIADH must be considered in every case with hyponatremia with low serum osmolality condition, a normal acid-base state, urine osmolality over 100 mOsm/kg, and urine sodium concentration more than 40 meq/L. Also, generalized or local infections are important and unregarded causes of SIADH. Multiple infectious diseases are associated with this syndrome (26).

In addition to the disease itself, some anti-TB drugs can also be round anemia. Nakashita et al. has reported a case of SIADH caused by ethionamide in a patient with PTB also they suggested that anti-TB drugs should be considered as the possible cause of SIADH but the result of this study was that the incidence of hyponatremia in patients who received ethionamide with maximum dose was not higher than those who received a lesser one (27).

Besides, non-tuberculoses pneumonia is a very rare cause of SIADH induced hyponatremia. Charles et al. described a patient who had lobar pneumonia presenting with confusion due to severe hyponatremia (28). Also, Rivers et al., expressed a child with excessive secretion of ADH in association with common pulmonary infections (29).

Endocrine system involvements by TB, as the other mechanism, can induce hyponatremia which is important to consider in patients with PTB. TB was revealed to involve adrenal glands directly (30) and this involvement lead to overt or subclinical adrenal insufficiency and hyponatremia (6). Pituitary gland may also be involved by the tuberculosis bacilli. Hypopituitarism has been reported in 20% of cases years after the treatment of tubercular meningitis in childhood. The reason seemed to be tuberculosis lesions impressing the hypothalamus, pituitary stalk and indirectly or directly, the pituitary gland itself (8).

The hyponatremia due to PTB is usually mild to moderate, asymptomatic, and self-limited. SIADH is commonly reversible with effective PTB treatment in most cases (25). Therefore, it can be overlooked if the physician does not give sufficient attention. On the other hand, patients who affected by hyponatremia were more likely to have higher mortality. Sharma et al. suggested hyponatremia as predictors of development and outcome in patients with acute respiratory distress syndrome due to tuberculosis (31).

Moreover, other electrolyte disturbances such as hypercalcemia have been reported as one of the most common electrolyte imbalance in 25.7% of patients with PTB although hyponatremia developed lower prevalence (22.15%)(32). Furthermore, the incidence of hyponatremia in patients with AIDS complicating with TB is higher. Smith et al. revealed that hyponatremia was discovered in 60% of AIDS patients with a diagnosis of generalized tuberculosis, however half of these patients of disseminated tuberculosis were only diagnosed after death. In our study, we overruled the HIV positive patients and it can be the reason of the differences between our findings and others.

In the present study, the higher age among the demographic characteristics was observed in patients with PTB and hyponatremia than has previously been noted. Few studies evaluated the role of age on presenting hyponatremia in patients with neural defects (33). The age range in mentioned reports was documented widespread and generally it is impossible to clearly the role of age on hyponatremia in patients with PTB. In addition, it should be suggested that hyponatremic patients should be evaluated for PTB when an initial investigation failed to test. We would recommend that patients with PTB (especially older age patients) should be closely observed for electrolyte imbalance. We also recommend further studies with a greater sample size mainly focused on the predisposing factors of electrolyte imbalance in patients suffering from PTB.
